# A New Strategy for High-Efficient Tandem Enrichment and Simultaneous Profiling of N-Glycopeptides and Phosphopeptides in Lung Cancer Tissue

**DOI:** 10.3389/fmolb.2022.923363

**Published:** 2022-05-24

**Authors:** Zhuokun Du, Qianying Yang, Yuanyuan Liu, Sijie Chen, Hongxian Zhao, Haihong Bai, Wei Shao, Yangjun Zhang, Weijie Qin

**Affiliations:** ^1^ School of Basic Medical Science, Anhui Medical University, Hefei, China; ^2^ State Key Laboratory of Proteomics, National Center for Protein Sciences Beijing, Beijing Institute of Lifeomics, Beijing Proteome Research Center, Beijing, China; ^3^ Phase I Clinical Trial Center, Beijing Shijitan Hospital of Capital Medical University, Beijing, China

**Keywords:** proteomics, enrichment, N-glycopeptides, phosphopeptides, mass spectrometry

## Abstract

N-glycosylation and phosphorylation, two common posttranslational modifications, play important roles in various biological processes and are extensively studied for biomarker and drug target screening. Because of their low abundance, enrichment of N-glycopeptides and phosphopeptides prior to LC–MS/MS analysis is essential. However, simultaneous characterization of these two types of posttranslational modifications in complex biological samples is still challenging, especially for tiny amount of samples obtained in tissue biopsy. Here, we introduced a new strategy for the highly efficient tandem enrichment of N-glycopeptides and phosphopeptides using HILIC and TiO_2_ microparticles. The N-glycopeptides and phosphosites obtained by tandem enrichment were 21%–377% and 22%–263% higher than those obtained by enriching the two PTM peptides separately, respectively, using 160–20 μg tryptic digested peptides as the starting material. Under the optimized conditions, 2798 N-glycopeptides from 434 N-glycoproteins and 5130 phosphosites from 1986 phosphoproteins were confidently identified from three technical replicates of HeLa cells by mass spectrometry analysis. Application of this tandem enrichment strategy in a lung cancer study led to simultaneous characterization of the two PTM peptides and discovery of hundreds of differentially expressed N-glycosylated and phosphorylated proteins between cancer and normal tissues, demonstrating the high sensitivity of this strategy for investigation of dysregulated PTMs using very limited clinical samples.

## Introduction

Protein posttranslational modifications (PTMs) have important effects on almost all aspects of protein functions, such as signal transduction, protein–protein interactions, enzyme activity and metabolic regulation (Beltrao et al., 2012; Wani et al., 2015; Wesseling et al., 2020). As two of the most common PTMs, protein N-glycosylation and phosphorylation play important roles in various biological processes (Brüning et al., 2019; Zhang Q. et al., 2020; Huang et al., 2021). Therefore, the study of N-glycosylation and phosphorylation is of great significance for understanding the mechanism of disease, identifying biomarkers and discovering drug targets (Cutillas, 2015; Bian et al., 2016; Sajic et al., 2018). The possibility of direct competition or indirect regulation between phosphorylation and N-glycosylation has been reported due to spatial competition among identical or adjacent modification sites (Song et al., 2021). In addition, abnormal N-glycosylation and phosphorylation modifications coexist in many disease-related proteins (He et al., 2022). For example, in Alzheimer’s disease, N-glycosylation of Tau promotes its phosphorylation and helps maintain its paired helical filament structure (Losev et al., 2021). Therefore, simultaneous characterization of these two PTMs may provide key information for protein function regulation. Currently, the most widely used tool for high-throughput PTM profiling is liquid chromatography tandem mass spectrometry (LC−MS/MS) (Aebersold and Mann, 2003; Zolg et al., 2018). However, the direct analysis of N-glycopeptides and phosphopeptides in complex samples is almost infeasible due to their low abundance, low ionization efficiency, and signal inhibition caused by massive unmodified peptides (Capriotti et al., 2018). Therefore, enrichment of N-glycopeptides and phosphopeptides prior to LC–MS/MS analysis is essential. In recent years, various N-glycopeptide enrichment approaches have been developed and widely used (Qi et al., 2021), including hydrazide chemistry (Bai et al., 2017), lectin affinity chromatography (Zielinska et al., 2010; Totten et al., 2018), borate affinity chromatography (Bie et al., 2015; Xie et al., 2018; Chen et al., 2021) and hydrophilic interaction liquid chromatography (HILIC) (Ahn et al., 2015; Zhan et al., 2019; Zhang H. et al., 2020). Among them, HILIC has become the most advantageous due to its unbiasedness toward N-glycopeptides with different glycans, applicability for intact N-glycopeptide enrichment and good compatibility with LC–MS/MS analysis (Cummings and Pierce, 2014; Qing et al., 2020). Likewise, in terms of enrichment strategies for phosphoproteome analysis, numerous methods have been developed and can be divided into the following four categories: ion-exchange and mixed-mode chromatography (Dong et al., 2010; Low et al., 2021b), affinity-based chromatography (Chen et al., 2016), antibody and protein domain-based enrichment of pTyr (Liu et al., 2022), and functionalized polymer (Zhang et al., 2019; Peng et al., 2020; Low et al., 2021a). Among them, metal oxide affinity chromatography (MOAC), represented by TiO_2_, has shown excellent enrichment performance and strong compatibility with the most commonly used buffers (Yang et al., 2017).

Commonly, N-glycopeptides and phosphopeptides are separately enriched using different enrichment methods (Wang et al., 2019). Separate enrichment requires larger amount of samples, which may not be available for clinical samples, such as tissue biopsy samples. Therefore, simultaneous enrichment of N-glycopeptides and phosphopeptides from the same sample is highly desired. However, due to the differences between the properties of N-glycopeptides and phosphopeptides (Kaasik et al., 2013; Wang et al., 2019), it is still challenging to find compatible methods capable of enriching both PTM peptides with maximized efficiency. As a result, coeluted N-glycopeptides, phosphopeptides and nonmodified peptides lead to inevitable interference and signal suppression and limit detection sensitivity (Huang et al., 2019). To overcome the above shortcomings, in this study, we developed a robust, simple enrichment method for the efficient simultaneous identification of N-glycopeptides and phosphopeptides from the same sample. In our strategy, ZIC-HILIC and TiO_2_ microparticles were employed sequentially for tandem enrichment of N-glycopeptides and phosphopeptides. 2798 N-glycopeptides from 434 N-glycoproteins and 5130 phosphosites from 1986 phosphoproteins were confidently identified from HeLa cells in three replicates. Compared with separate enrichment of the two PTM peptides, N-glycopeptides and phosphosites obtained by our tandem enrichment were 21%–377% and 22%–263% higher, respectively, using 160–20 μg tryptic digested peptides as the starting material. Compared with the previous report for enrichment of N-glycopeptides and phosphopeptides (Silbern et al., 2021), our final optimized protocol is simpler and with higher enrichment efficiency. Due to the higher compatibility of the buffers used for N-glycopeptide and phosphopeptide enrichment in our tandem enrichment method, the flow-through and washing solution of N-glycopeptide enrichment can be directly used for phosphopeptide enrichment without introducing extra drying step or buffer exchange. Therefore, reduced loss of peptides, and higher enrichment efficiency can be expected by our method. Furthermore, since no salt buffer is introduced in our method, no desalting step is required, which further improves the recovery rate. Therefore, our method is more suitable for micro-samples. Finally, using the same amount of peptides, our method requires 87.5% less HILIC materials and 37.5% less TiO_2_ materials and therefore is more suitable for analyzing large-scale of clinical samples.

Abnormal N-glycosylation and phosphorylation of proteins are closely related to the occurrence and development of many diseases, especially cancer (Zhu et al., 2019; Mahoney et al., 2021). In recent years, many studies have found N-glycoproteins and phosphoproteins as potential biomarkers or drug targets for cancer (Wang et al., 2020; Lin et al., 2021). Our tandem enrichment strategy was applied in a lung cancer study and led to the discovery of 249 and 486 differentially expressed N-glycosylated and phosphorylated proteins between cancer and normal tissues, respectively. The above results demonstrated the advantages of our strategy for simultaneous characterization of the two PTM peptides using very limited sample amount, as is often the case for tissue biopsy studies.

## Materials and Methods

### Sample Preparation and Protein Extraction

HeLa cells were cultured in Dulbecco’s modified Eagle’s medium (DMEM, Gibco, United Kingdom) supplemented with 10% (v/v) fetal bovine serum (FBS, Gibco, United Kingdom), 100 U/mL penicillin and 100 mg/ml streptomycin (HyClone, United States) and incubated at 37°C in a 5% CO2 humidified incubator. Once 80% confluency was achieved, the supernatant was carefully removed, and the cells were washed twice with PBS. Then, the HeLa cells were lysed using 8 M urea lysis buffer (0.1% v/v SDS, 8 M urea, and 100 mM Tris-HCl, pH = 8). The cell samples in the lysis buffer were continually sonicated in a probe sonicator with 20% energy and pulsed 2 s on 2 s off for 99 cycles. The homogenates were centrifuged at 16,000 g at 4°C for 15 min, and the supernatants were collected.

Thirty mg lung cancer tissue and normal tissue were weighed and washed with PBS several times to remove blood residue. Then, the sample was transferred to a 2 ml tube with 0.5 mm ceramic beads. Then, 300 μL lysis solution (0.1% v/v SDS, 8 M urea, and 100 mM Tris-HCl, pH = 100) containing protease and phosphatase inhibitors (Thermo Fisher, United States) was added to the tube. Tissue homogenization using an Omni Bead Ruptor Elite (OMNI International, Inc., Kennesaw, GA, United States) was conducted for 30 s at 6 m/s. The resulting homogenate was then centrifuged at 16,000 g for 15 min, and the supernatant was collected.

### Protein Digestion

The protein lysates were processed by filter-aided sample preparation (FASP) (Wisniewski et al., 2009). Then, 0.1 M DTT in 8 M urea was added to the protein lysates to a final concentration of 10 mM DTT. The protein lysates were placed at 37°C for 4 h, pipetted into Microcon 30k centrifugal ultrafiltration units and centrifuged at 14,000 g for 100 min. Then, 200 μL of 50 mM CAA in UA (8 M urea, and 100 mM Tris-HCl, pH = 100) was added to the filters, and the samples were incubated for 40 min. The filters were washed with 200 μL of UA followed by three washes with 200 μL of 50 mM NH_4_HCO_3_ (ABC). Then, the protein lysates were digested in 200 μL of ABC at 37°C for 16 h using trypsin at an enzyme to protein ratio of 1:100. The digested peptides were collected by centrifugation at 14,000 *g* for 10 min followed by two washes with 200 μL of deionized water. The digested samples were dried in a SpeedVac and stored at −80°C for later use.

### Enrichment of N-Glycopeptides

ZIC-HILIC microparticles (5 μm, 200 Å, Merck, Germany) were weighed at a fixed peptide to ZIC-HILIC ratio (the final optimized peptide to ZIC-HILIC ratio was 160 μg: 1 mg). ZIC-HILIC microparticles were washed with 80% v/v ACN and 1% v/v TFA (binding buffer 1). The ZIC-HILIC microparticles resuspended in 100 μL deionized water were loaded into a 200 μL tip. The tip was prepared by capping at the end with two layers of C_8_ film. Then, 50 μL deionized water was added to the tip, and it was centrifuged at 6000 rpm for 2 min. Then, 50 μL binding buffer 1 was added to the tip, and it was centrifuged at 3,000 rpm for 3 min twice. The tryptic digests were dissolved in 50 μL binding buffer 1, loaded into the tip and centrifuged at 3,000 rpm for 3 min. The flow-through was reloaded into the tip and centrifuged at 4,000 rpm for 3 min. The ZIC-HILIC microparticle-bound N-glycopeptides were washed twice with 200 μL binding buffer 1. Then, the bound N-glycopeptides were eluted three times with 50 μL eluting buffer (20% v/v ACN, 1% v/v TFA), freeze dried, and dissolved in 0.1% v/v FA for LC–MS/MS analysis.

### Enrichment of Phosphopeptides

TiO_2_ microparticles (5 μm, GL Sciences, Japan) were weighed at a fixed peptide to TiO_2_ ratio (the final optimized peptide to TiO_2_ ratio was 160 μg: 1 mg). The TiO_2_ microparticles were washed with washing buffer (80% v/v ACN, 1% v/v TFA) and binding buffer 2 (80% v/v washing buffer, 20% v/v lactic acid). The tryptic digests were dissolved in binding buffer 2 and mixed with the TiO_2_ microparticles. The mixture was incubated for 1 h and then loaded into a 200 μL tip. The tips were prepared by capping at the end with two layers of C_8_ film. The tips were centrifuged at 1000 g for 10 min, and then the flow-through was reloaded into the tip, and it was centrifuged at 1,000 g for 10 min. The TiO_2_-bound phosphopeptides were washed with 200 μL binding buffer 2 twice and 200 μL washing buffer four times. Then, the bound phosphopeptides were eluted with 200 μL eluting buffer (15% v/v NH_3_H_2_O) twice, freeze dried, and dissolved in 0.1% v/v FA for LC–MS/MS analysis.

### Tandem Enrichment of N-Glycopeptides and Phosphopeptides

The peptide samples were dissolved in 50 μL binding buffer 1 to enrich N-glycopeptides using the method described above. The flow-through of loading and washing buffer obtained in N-glycopeptide enrichment was collected and combined. Next, lactic acid was added to the mixture to make a final concentration of 20% v/v for phosphopeptide enrichment using the method described above.

### LC–MS/MS Analysis

A Q-Exactive HF mass spectrometer equipped with an EASY-nLC 1200 nano-LC system (Thermo Fisher Scientific, United States) was used for LC–MS analysis. The samples were dissolved in 0.1% FA and loaded into a 15 cm length reversed-phase column (150 nm id) packed with Ultimate XB-C18 1.9 μm resin (Welch materials). The constant flow rate was 600 nL/min. The eluted peptides were analyzed by data-dependent MS2 acquisition (DDA). Higher-energy collision dissociation (HCD) with a normalized collision energy of 27% was used for peptide fragmentation.

### Data Analysis

For identification of intact N-glycopeptides, the raw files were searched against Byonic software (Protein Metrics, San Carlos, CA) with a precursor ion mass tolerance of 10 ppm and a fragment ion tolerance of 0.02 Da. Acetylation of protein N-terminus and the oxidation of methionine (M) were set as variable modifications. Meanwhile, the carbamidomethylation of cysteine (C) was set as a fixed modification. N-glycopeptide searching was conducted using a N-glycome database that contained 156 glycans. The protein FDR cutoff was 1%. Strict filtering of N-glycopeptides was applied using a Byonic score cutoff >300. For the identification of phosphopeptides, the raw files were searched against MaxQuant software with a precursor ion mass tolerance of 4.5 ppm and a fragment ion tolerance of 20 ppm. The PSM FDR cutoff and protein FDR cutoff were both set at 1%. Phosphorylation of serine (S), threonine (T), and tyrosine (Y), oxidation of methionine (M) and acetylation of protein N-terminus were set as variable modifications. Meanwhile, carbamidomethylation of cysteine (C) was set as a fixed modification. Phosphosites with a localization probability of >0.75 were accepted as confident identifications.

## Results

**FIGURE 1:**
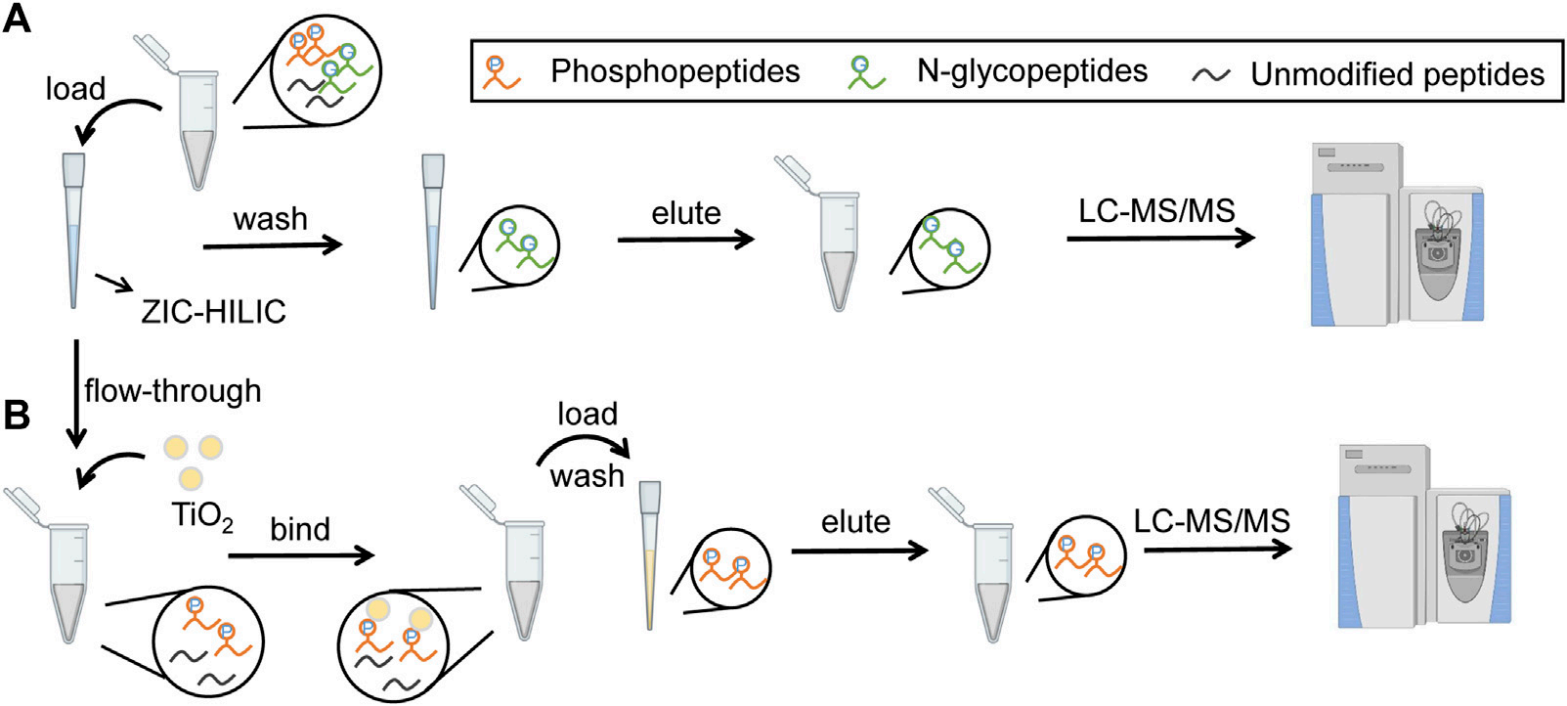
Workflow of tandem enrichment of N-glycopeptides and phosphopeptides. **(A)** enrichment of N-glycopeptides. **(B)** enrichment of phosphopeptides.

In our strategy, N-glycopeptides and phosphopeptides from the same samples were tandemly enriched by ZIC-HILIC microparticles and TiO_2_ microparticles, as shown in Figures 1A,B. When loading samples into the tip packed with ZIC-HILIC microparticles, N-glycopeptides were retained in the tip, while phosphopeptides and unmodified peptides flowed through the tip. The retained N-glycopeptides were then eluted from the ZIC-HILIC microparticles and collected. Next, the phosphopeptides and unmodified peptides were added into the tip packed with TiO_2_ microparticles. After removal of the unmodified peptides, the remaining phosphopeptides were eluted and collected. Through our strategy, N-glycopeptides and phosphopeptides from the same sample can both be enriched using obviously reduced sample amounts. Therefore, this strategy is particularly suitable for clinical applications using limited sample amounts, such as tissue biopsy.

### Optimization of Enrichment of N-Glycopeptides

**FIGURE 2:**
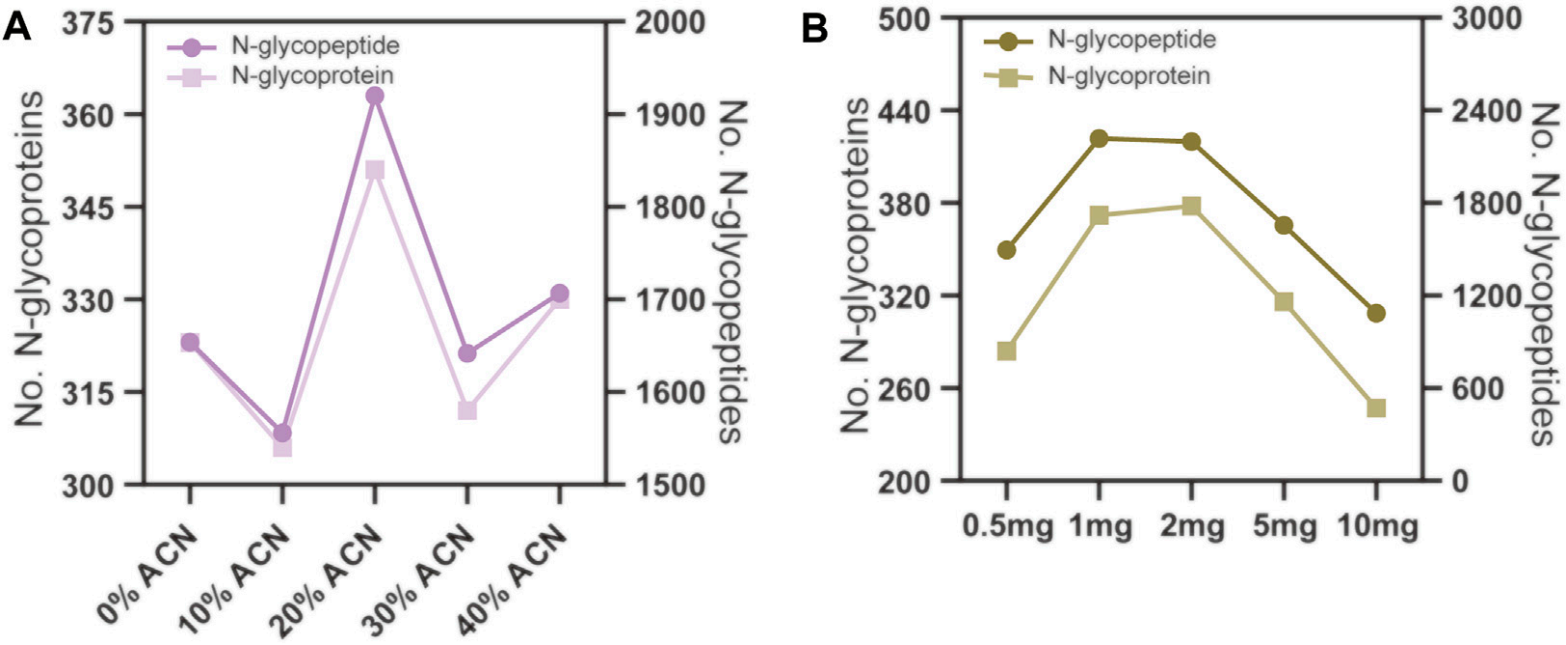
N-glycopeptide enrichment from 160 μg peptide samples using different conditions. **(A)** the ACN volume ratio in the eluting buffer were 0%, 10%, 20% 30% and 40%. **(B)** the amount of ZIC-HILIC microparticles were 0.5, 1, 2, 5 and 10 mg.

The conditions for N-glycopeptide and phosphopeptide enrichment were first separately optimized to achieve the maximum identification efficiency of the PTM peptides. To evaluate the effect of different ACN concentrations in the eluting buffers on N-glycopeptide enrichment by ZIC-HILIC microparticles, 160 μg peptide samples were used for N-glycopeptide enrichment. The ACN concentrations in the eluting buffer were 0%, 10%, 20%, 30% and 40%, v/v. The enrichment products were analyzed by LC–MS/MS and searched by Byonic software. As shown in Figure 2A and Supplementary Table S1, the N-glycopeptide identification result showed that eluting buffer containing 20% ACN is the most efficient condition to elute N-glycopeptides from the ZIC-HILIC microparticles, with 1920 N-glycopeptides and 351 N-glycoproteins identified.

Next, we evaluated the effect of the peptide to ZIC-HILIC microparticle ratio on N-glycopeptide enrichment. The peptide samples used were fixed at 160 μg, and the amounts of ZIC-HILIC microparticles were 0.5, 1, 2, 5 and 10 mg. As shown in Figure 2B and Supplementary Table S1, 1 mg ZIC-HILIC microparticles resulted in the highest number of identified N-glycopeptides among the tested conditions. Therefore, a 160 μg: 100 mg peptide to ZIC-HILIC microparticle ratio was adopted in the subsequent tests.

### Optimization of Enrichment of Phosphopeptides

**FIGURE 3:**
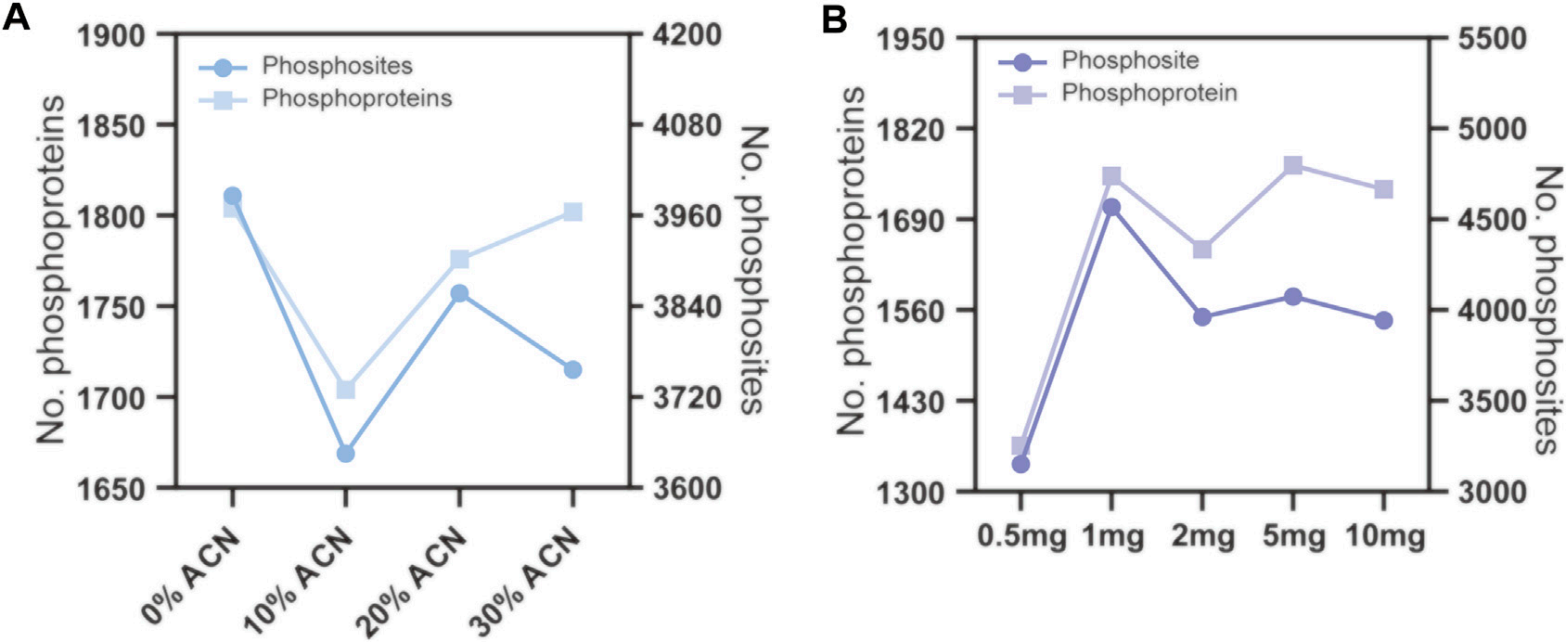
Phosphopeptide enrichment from 160 μg peptide samples using different conditions. **(A)** the ACN volume ratio of the eluting buffer were 0%, 10%, 20% and 30%. **(B)** the amount of TiO_2_ microparticles were 0.5, 1, 2, 5 and 10 mg.

To evaluate the effect of the concentration of ACN in the eluting buffers on phosphopeptide enrichment by TiO_2_ microparticles, 160 μg peptide samples were used for phosphopeptide enrichment, and the ACN concentrations in the eluting buffer were 0%, 10%, 20% and 30%, v/v. The enrichment products were analyzed by LC–MS/MS and searched by MaxQuant software. As shown in Figure 3A and Supplementary Table S2, since 0% ACN results in the most phosphosites and phosphoproteins identified, this condition was chosen for further optimization. Next, we evaluated the effect of the peptide to TiO_2_ ratio on phosphopeptide enrichment. The peptide samples used were fixed at 160 μg, and the amount of TiO_2_ microparticles was 0.5, 1, 2, 5 and 10 mg. As shown in Figure 3B and Supplementary Table S2, the number of identified phosphoproteins and phosphosites first increased when the amount of TiO_2_ increased from 0.5 to 1 mg and then reached a plateau. No further enhancement was found for 2, 5 and 10 mg TiO_2_. According to the above results, eluting buffer without ACN and a peptide to TiO_2_ ratio of 160 μg:1 mg were adopted for subsequent phosphopeptide enrichment.

### Tandem Enrichment of N-Glycopeptides and Phosphopeptides

After optimizing the conditions used in separate enrichment of N-glycopeptides and phosphopeptides, the feasibility of tandem enrichment of the two PTM peptides from the same sample was evaluated. To tandemly enrich N-glycopeptides and phosphopeptides, the flow-through of loading and washing solutions of the first enrichment need to be collected and used in the second enrichment. To decide which PTM peptide should be enriched first, we analyzed the buffer conditions of the two enrichment methods. The main difference in the N-glycopeptide and phosphopeptide enrichment methods was the binding buffer. The binding buffer for N-glycopeptide enrichment was 80% v/v ACN, 1% v/v TFA and 19% water (binding buffer 1), while that for phosphopeptide enrichment was 80% v/v binding buffer 1 and 20% lactic acid. If phosphopeptides are enriched first, the flow-through of loading and washing solutions contains lactic acid, which is not compatible with N-glycopeptide enrichment. We found that 20% lactic acid in the loading buffer led to an obvious decrease in the enrichment of N-glycopeptides, with only 43% identified N-glycopeptides using the same amount of starting peptides. Therefore, we chose to enrich N-glycopeptides first in our tandem enrichment method, and the flow-through of loading and washing solutions of the N-glycopeptide enrichment was used to enrich phosphopeptides.

**FIGURE 4:**
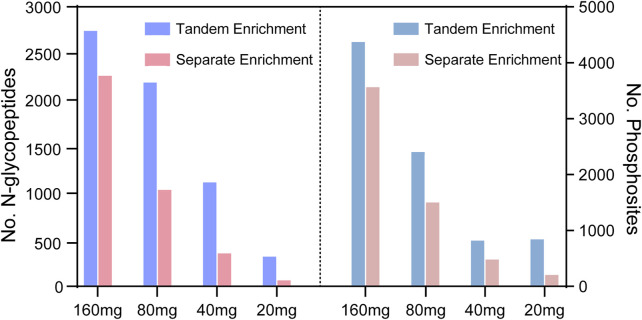
N-glycopeptides and phosphosites obtained from tandem enrichment and separate enrichment of using 160, 80, 40 and 20 μg peptides.

To evaluate the performance of tandem enrichment, we used 160, 80, 40 and 20 μg peptide samples to tandemly enrich N-glycopeptides and phosphosites. The results were compared with those obtained using separate enrichment of the two PTM peptides. For separate enrichment, the starting peptides were divided into two halves for each PTM peptide enrichment. As shown in Figure 4, Supplementary Tables S3, S4 tandem enrichment identified 21%–377% more N-glycopeptides and 22%–263% more phosphosites than separate enrichment using 160–20 μg of peptide samples. Although the number of identified N-glycopeptides and phosphosites decreased with the decreasing amount of starting peptide sample, the advantage of using tandem enrichment was more obvious with a lower amount of starting peptides. When 20 μg of peptides was used as the starting material, tandem enrichment exhibited 3.7- and 2.6-fold enhancement compared with the number of N-glycopeptides and phosphosites identified by separate enrichment.

**FIGURE 5:**
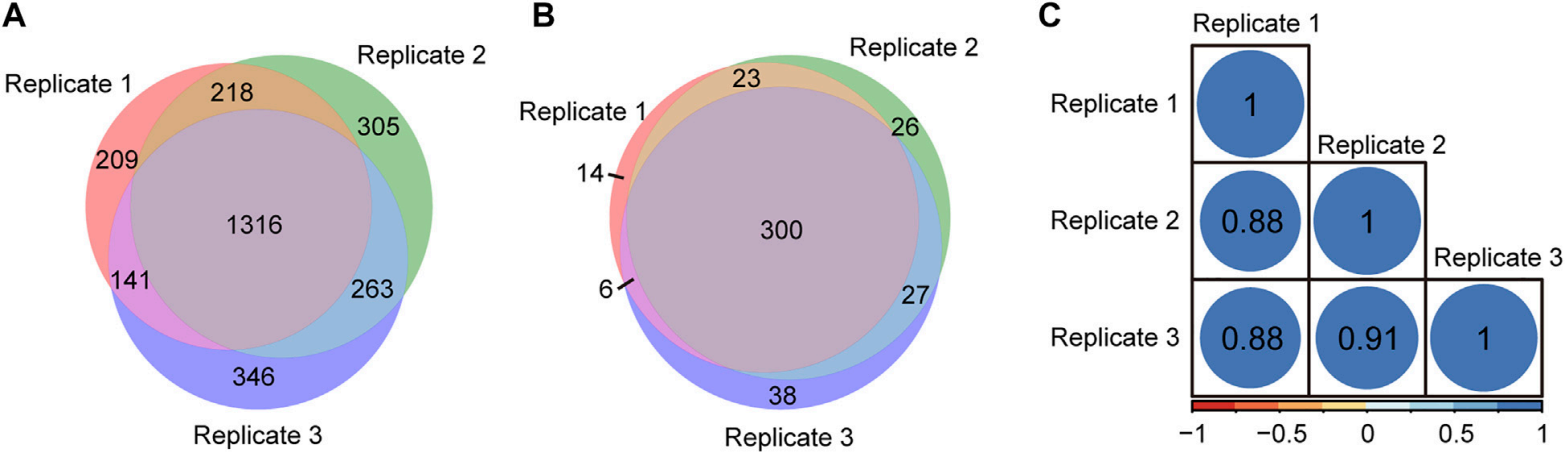
N-glycopeptides and N-glycoproteins identified from three replicates of tandem enrichment. **(A)** Identification reproducibility of N-glycopeptides. **(B)** Identification reproducibility of N-glycoproteins. **(C)** Quantification reproducibility of N-glycopeptides

**FIGURE 6:**
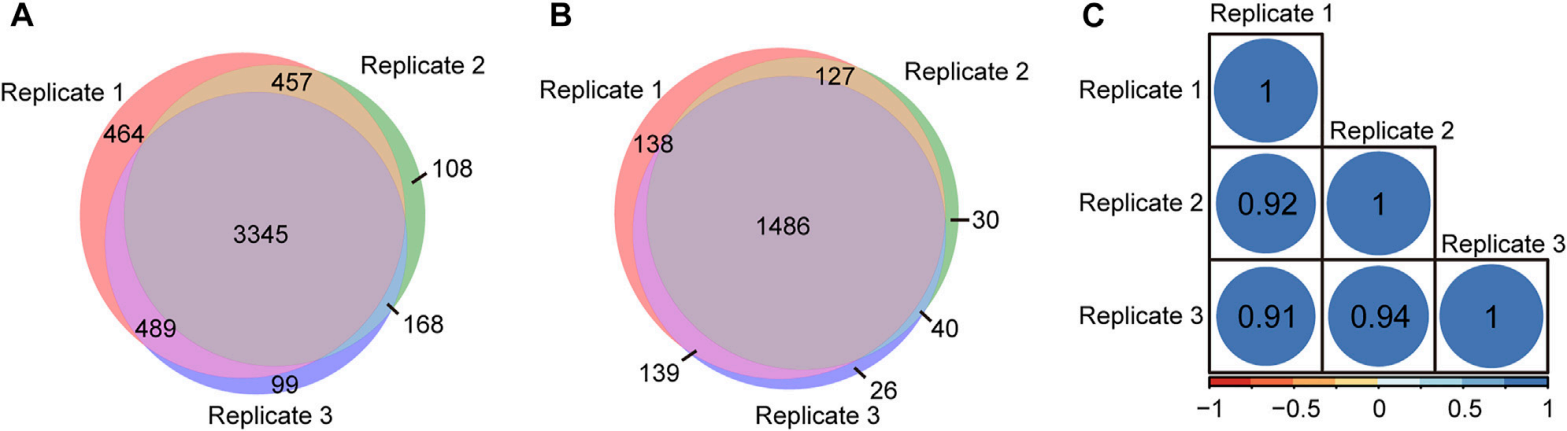
Phosphosites and phosphoproteins identified from three replicates of tandem enrichment. **(A)** Identification reproducibility of phosphosites. **(B)** Identification reproducibility of phosphoproteins. **(C)** Quantification reproducibility of phosphosites.

To evaluate the repeatability of tandem enrichment, three technical replicate tests were conducted using 160 μg peptide samples. The identification overlap and quantification correlation of the N-glycopeptides and phosphosites in the three tests are shown in Figure 5 and Supplementary Table S5. For N-glycopeptides, 2798 N-glycopeptides from 434 N-glycoproteins were obtained in three replicates, with 69% N-glycopeptides and 82% N-glycoproteins identified in at least two tests (Figures 5A,B). The Pearson correlation for N-glycopeptide quantification in three replicates was close to 0.9 (Figure 5C). For phosphopeptides (Figure 6 and Supplementary Table S6), 5130 phosphosites from 1986 phosphoproteins were obtained in three replicates, and 86% phosphosites (Figure 6A) and 89% phosphoproteins (Figure 6B) were identified in at least two replicates with a quantification correlation >0.9 (Figure 6C). The above results demonstrated good identification and quantification reproducibility for both N-glycopeptide and phosphopeptide enrichment.

### Differential Analysis of Phosphopeptides and N-Glycopeptides in Lung Cancer and Normal Tissue Using Tandem Enrichment

**FIGURE 7:**
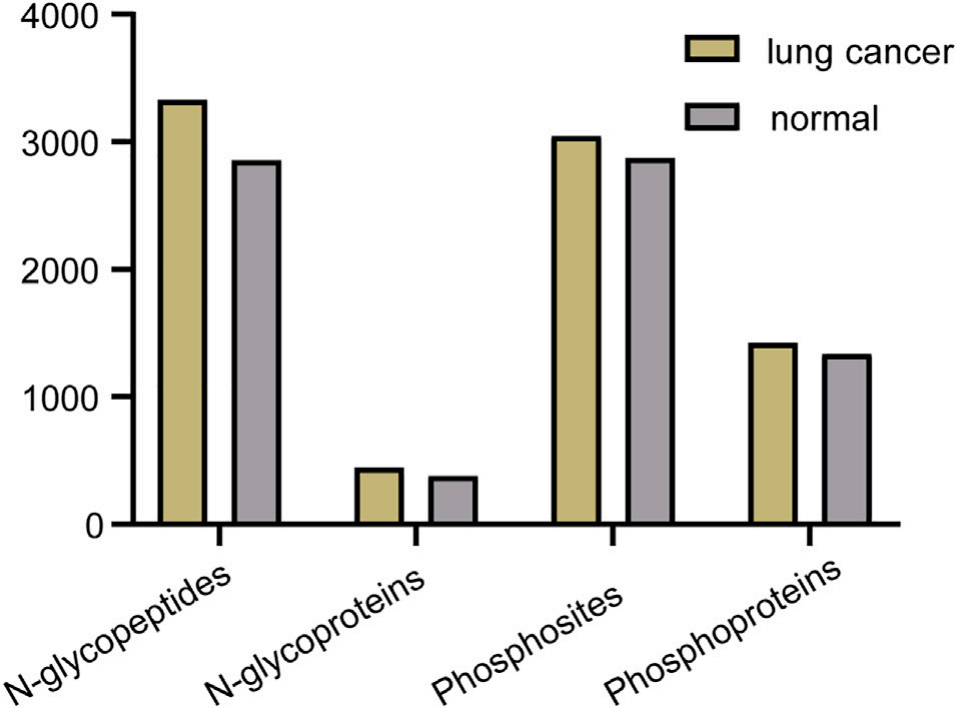
Number of N-glycopeptides, N-glycoproteins, phosphosites and phosphoproteins identified in three replicates in lung cancer and normal tissue.

Abnormal and dysregulated protein N-glycosylation and phosphorylation have been extensively reported in tumor tissues; therefore, deep profiling and identification of the key PTM proteins and modification sites are of particular importance for screening drug targets. Glycoproteins produced by cancer cells have altered glycan structures, although the proteins themselves are common (Narimatsu et al., 2010), and phosphorylation also plays a crucial role during cancer progression (Kitano et al., 2014). For the differential study of protein N-glycosylation and phosphorylation in lung cancer and normal tissue, N-glycopeptides and phosphopeptides were analyzed using our tandem enrichment strategy. Three replicate enrichments were performed for the two types of tissue. In total, we obtained 4847 N-glycopeptides, 540 N-glycoproteins, 3224 phosphosites and 1,488 phosphoproteins in three replicates. Lung cancer tissue displayed 6%–17% more modified proteins and 5–16% more modified peptides than normal tissue for both N-glycosylation and phosphorylation (Figure 7, Supplementary Tables S7, S8).

**FIGURE 8:**
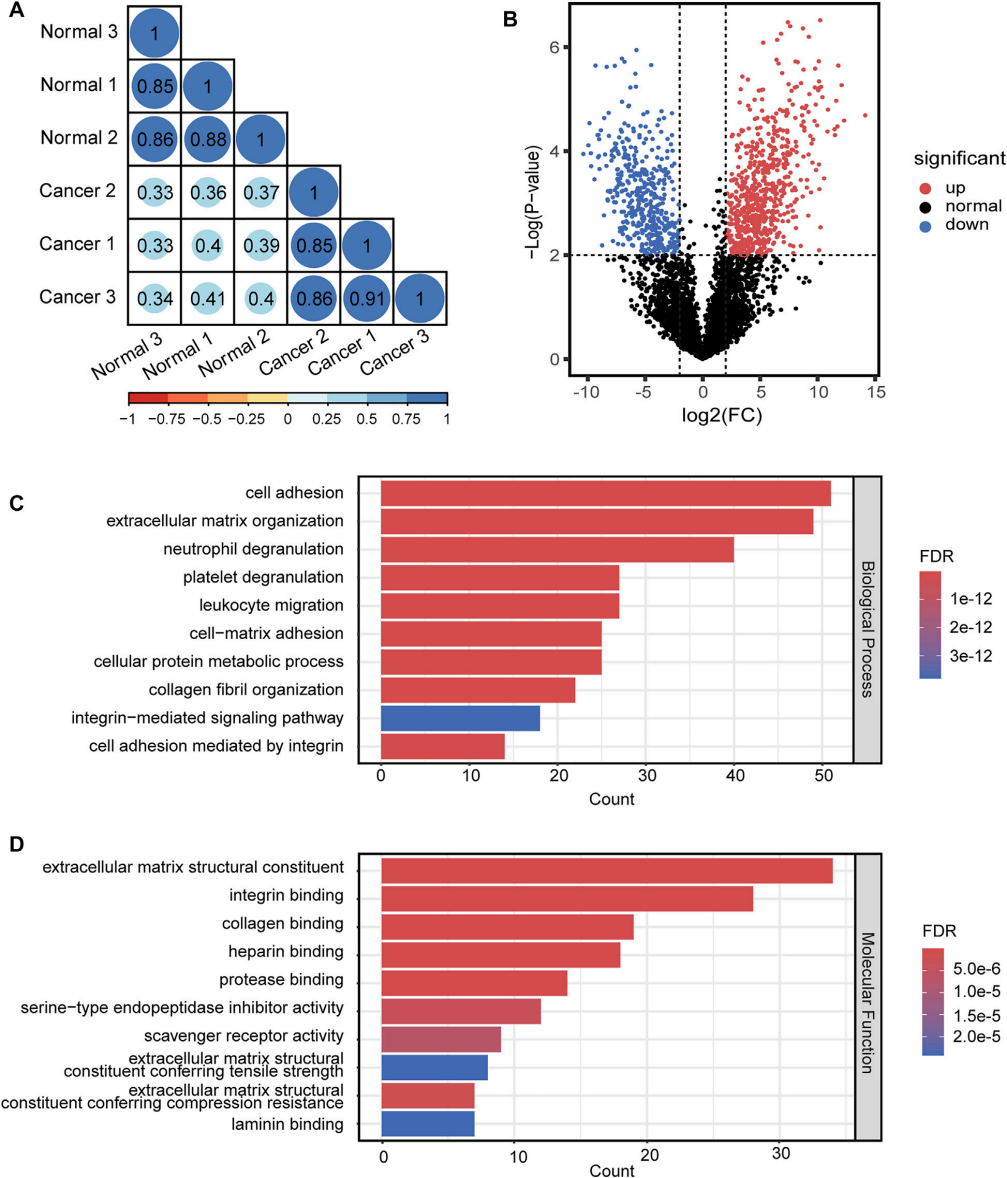
Quantitative differential analysis and GO analysis of N-glycopeptides from lung cancer and normal tissue. **(A)** quantification correlation of N-glycopeptides. **(B)** volcano plot of N-glycopeptides. **(C)** biological process of GO analysis of the dysregulated N-glycoproteins. **(D)** molecular function of GO analysis of the dysregulated N-glycoproteins.

**FIGURE 9:**
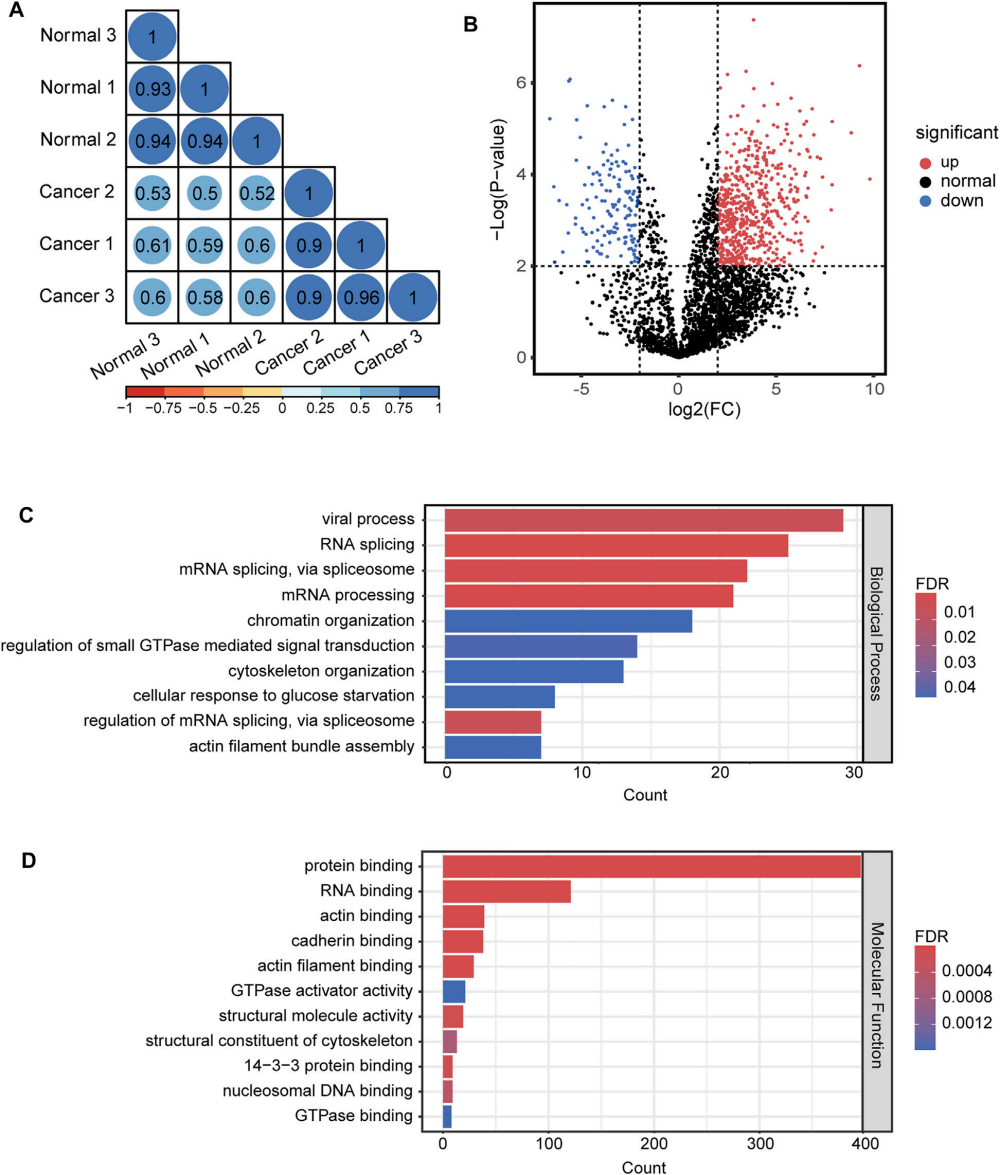
Quantitative differential analysis and GO analysis of phosphosites from lung cancer and normal tissue. **(A)** quantification correlation of phosphosites. **(B)** volcano plot of phosphosites. **(C)** biological process of GO analysis of the dysregulated phosphoproteins. **(D)** molecular function of GO analysis of the dysregulated phosphoproteins.

For quantitative analysis of N-glycopeptides/N-glycoproteins of the lung cancer and normal tissue, the Pearson correlation coefficients for replicated tests within the cancer tissue and normal tissue were all over 0.85, again demonstrating good quantification reproducibility of the tandem enrichment method. In contrast, the Pearson correlation coefficient between the cancer tissue and normal tissue was mostly below 0.4, indicating an obvious difference between the two types of samples (Figure 8A). The differentially expressed N-glycopeptides are further revealed in the volcano plot in Figure 8B and Supplementary Table S9. A total of 1,151 differentially expressed N-glycopeptides were obtained by filtering the abundance ratio (>4-fold) and *p* value (<0.01). On the basis of these N-glycopeptides, 249 up and down regulated N-glycoproteins were obtained in lung cancer. GO analysis of the dysregulated N-glycoproteins in the lung cancer tissue is shown in Figures 8C,D and Supplementary Table S9. In the biological process category, cell adhesion, extracellular matrix organization and neutrophil degranulation were the top enriched terms. In the molecular function category, extracellular matrix structural constituent, integrin binding and collagen binding were the top enriched terms. Furthermore, the dysregulated N-glycoproteins and GO functions found in this work were cross-referenced with the literature, and some of them were previously reported to be associated with the occurrence and development of cancer, indicating the potential of our tandem enrichment strategy for screening new drug targets for cancer treatment. For example, abnormal expression of folate receptor alpha (FRA) in different non-small-cell lung cancer (NSCLC) histological subtypes was previously reported, which may be associated with disease stage or survival in lung adenocarcinoma, suggesting the potential of FRA and its N-glycosylation to be a useful diagnostic and prognostic marker (Iwakiri et al., 2008; Li et al., 2012). Similarly, quantitative analysis of the phosphosites identified in the lung cancer and normal tissues revealed good reproducibility within the cancer tissue and normal tissue samples (Pearson correlation coefficient >0.9). The Pearson correlation coefficients of the phosphosites between the cancer tissue and normal tissue samples were all below 0.65 (Figure 9A), indicating higher consistency of protein phosphorylation between the two types of samples than N-glycosylation. A total of 799 differential phosphosites were screened by filtering the abundance ratio (>4-fold) and *p* value (<0.01), corresponding to 486 differential phosphoproteins (Figure 9B and Supplementary Table S10). The top enriched GO terms of the dysregulated phosphoproteins in the biological process category were viral processing, RNA splicing and mRNA splicing via spliceosome (Figure 9C and Supplementary Table S10). In the molecular function category, protein binding, RNA binding and actin binding were among the top enriched GO terms (Figure 9D and Supplementary Table S10). The above results indicated that abnormal protein phosphorylation in RNA binding and mRNA splicing were highly enriched in our sample and therefore providing potential targets for lung cancer treatment.

## Conclusion

In this work, we developed a new strategy for the simultaneous analysis of N-glycopeptides and phosphopeptides using tandem enrichment with HILIC and TiO_2_ microparticles. This strategy exhibited obviously enhanced N-glycopeptide and phosphopeptide enrichment and identification compared with the separate enrichment method. 2798 N-glycopeptides from 434 N-glycoproteins and 5130 phosphosites from 1986 phosphoproteins were confidently identified from 160 μg HeLa cell lysate with good identification and quantification reproducibility. Applying this strategy to simultaneously characterize the two PTMs in lung cancer and normal tissue resulted in the discovery of 249 and 486 dysregulated N-glycoproteins and phosphoproteins, respectively, that have a close correlation with cell adhesion and RNA splicing. The above results indicated the potential of our tandem enrichment strategy for clinical application with a limited sample amount.

## Data Availability

The datasets presented in this study can be found in online repositories. The names of the repository/repositories and accession number(s) can be found below: http://www.proteomexchange.org/, PXD033282.
