# Supplementary material for: A New Strategy for High-Efficient Tandem Enrichment and Simultaneous Profiling of N-Glycopeptides and Phosphopeptides in Lung Cancer Tissue
Source: Front Mol Biosci. 2022 May 24;9:923363. doi: 10.3389/fmolb.2022.923363 (PMC9171396; doi:10.3389/fmolb.2022.923363)
Supplement: Supplementary file 11 [file DataSheet1.docx]

**Description of Additional Supplementary Files**

**Supplementary Table 1**

**Description:** N-glycopeptide enrichment from 160 μg peptide samples using different conditions.

**Supplementary Table 2**

**Description:** Phosphopeptide enrichment from 160 μg peptide samples using different conditions.

**Supplementary Table 3**

**Description:** N-glycopeptides obtained from tandem enrichment and separate enrichment of using 160 μg, 80 μg, 40 μg and 20 μg peptides.

**Supplementary Table 4**

**Description:** Phosphosites obtained from tandem enrichment and separate enrichment of using 160 μg, 80 μg, 40 μg and 20 μg peptides.

**Supplementary Table 5**

**Description:** N-glycopeptides and N-glycoproteins identified from three replicates of tandem enrichment.

**Supplementary Table 6**

**Description:** Phosphopeptide enrichment from three replicates of tandem enrichment.

**Supplementary Table 7**

**Description:** N-glycopeptide enrichment from three replicates of lung cancer and normal tissue.

**Supplementary Table 8**

**Description:** Phosphopeptide enrichment from three replicates of lung cancer and normal tissue.

**Supplementary Table 9**

**Description:** Quantitative differential analysis and GO analysis of N-glycopeptides from lung cancer and normal tissue.

**Supplementary Table 10**

**Description:** Quantitative differential analysis and GO analysis of phosphosites from lung cancer and normal tissue.
